# L-BSE experimentally transmitted to sheep presents as a unique disease phenotype

**DOI:** 10.1186/s13567-016-0394-1

**Published:** 2016-11-08

**Authors:** Marion M. Simmons, Melanie J. Chaplin, Timm Konold, Cristina Casalone, Katy E. Beck, Leigh Thorne, Sharon Everitt, Tobias Floyd, Derek Clifford, John Spiropoulos

**Affiliations:** 1 Department of Pathology, APHA Weybridge, Woodham Lane, Addlestone, Surrey, KT15 3NB UK; 2 Animal Sciences Unit, APHA Weybridge, Woodham Lane, Addlestone, Surrey, KT15 3NB UK; 3 Istituto Zooprofilattico Sperimentale del Piemonte, Liguria e Valle d’Aosta Sede Centrale di Torino, via Bologna, 148, 10154 Turin, Italy; 4 Department of Virology, APHA Weybridge, Woodham Lane, Addlestone, Surrey, KT15 3NB UK

## Abstract

Apart from prion protein genotype, the factors determining the host range and susceptiblity for specific transmissible spongiform encephalopathy agents remain unclear. It is known that bovine atypical L-BSE can transmit to a range of species including primates and humanised transgenic mice. It is important, therefore, that there is as broad an understanding as possible of how such isolates might present in food animal species and how robust they are on inter- and intra-species transmission to inform surveillance sytems and risk assessments. This paper demonstrates that L-BSE can be intracerebrally transmitted to sheep of several genotypes, with the exception of ARR/ARR animals. Positive animals mostly present with a cataplectic form of disease characterized by collapsing episodes and reduced muscle tone. PrP accumulation is confined to the nervous system, with the exception of one animal with lymphoreticular involvement. In Western blot there was maintenance of the low molecular mass and glycoform profile associated with L-BSE, irrespective of ovine host genotype, but there was a substantially higher N-terminal antibody signal relative to the core-specific antibody, which is similar to the ratio associated with classical scrapie. The disease phenotype was maintained on experimental subpassage, but with a shortened survival time indicative of an original species barrier and subsequent adaptation. Passive surveillance approaches would be unlikely to identify such cases as TSE suspects, but current statutory active screening methods would be capable of detecting such cases and classifying them as unusual and requiring further investigation if they were to occur in the field.

**Electronic supplementary material:**

The online version of this article (doi:10.1186/s13567-016-0394-1) contains supplementary material, which is available to authorized users.

## Introduction

The transmissible spongiform encephalopathies (TSE), fatal neurodegenerative diseases of animals, have been recognised for nearly three hundred years. Despite similar diseases occurring in man (e.g. [1]) the animal TSE were not regarded as zoonotic until the emergence in 1996 of variant Creutzfeldt-Jakob Disease (vCJD), linked to bovine spongiform encephalopathy (BSE) [2–4] which was first described in cattle in the 1980s [5]. The subsequent BSE epidemic, driven by the recycling of the agent in feedstuffs, affected nearly 200 000 cattle in the UK and, to a lesser extent, elsewhere, particularly in Europe [6]. It is thought to have been attributable to a single strain of agent [7–9], now referred to as classical BSE (C-BSE).

Following the implication of BSE as the origin of vCJD in man, substantial effort and expense has gone into ensuring the safety of the animal feed and human food chains. It was established through experimental challenge that sheep and goats were susceptible to C-BSE [10, 11] and a formal component of disease surveillance currently requires the classification of all TSE positive small ruminant isolates as “BSE-like” or “non-BSE-like” [EC TSE surveillance regulations (999/2001 as amended 36/2005)]. This reflects the hypothetical risk that would have been posed to the sheep population through exposure to BSE-contaminated concentrate feeds prior to the banning of mammalian protein in mammalian feedstuffs. These concerns have since been reinforced by the identification of two naturally-occurring cases of classical BSE in goats, one in France [12] and one in Scotland [13, 14].

Since its introduction in 2001, systematic EU-wide active surveillance for TSE in cattle and small ruminants [EU reg 999/2001] has resulted in the detection of two additional forms of BSE in cattle, commonly referred to collectively as “atypical”, that affected mainly cattle eight years of age or older (for reviews see [15, 16]). These cases were characterised as different from C-BSE, and designated H-BSE and L-BSE (also referred to as bovine amyloidotic spongiform encephalopathy (BASE) [17]), based on molecular features of the disease-associated form (PrP^Sc^) of the host PrP, or prion protein, which is the marker recognised by all current surveillance tests [18, 19]. To date, none of the “atypical” BSE cases diagnosed in various countries in cattle (*Bos taurus*) have been reported as clinical TSE suspects, and most cases have been identified through the surveillance of fallen stock. Given the rarity of these cases, and their widespread geographical distribution it has been speculated, but not yet established, that these atypical forms of disease are genetic in origin and/or arise spontaneously, and that they may be the origin of classical BSE [20, 21]. Even if there is no evidence of natural spread between animals in the field, these variants have been shown (like C-BSE) to transmit experimentally into a range of host species.

There is still no robust understanding of what makes a particular host susceptible, or a TSE isolate zoonotic [22], and it has been demonstrated that atypical BSE can transmit to a range of species including primates [23–25] and humanised transgenic mice [26, 27]. It is important, therefore, that there is as broad an understanding and awareness of how such isolates present in food animal species, how robust they are on inter- and intra-species transmission, and that they are characterised as fully as possible to assist with the maintenance of robust surveillance sytems and the assessment of risk to both human and animal health.

The susceptibility of sheep to scrapie and BSE is strongly influenced by different polymorphisms of the *PRNP* gene that encodes for prion protein (PrP), with polymorphisms at codons 136 (A or V), 141 (L or F), 154 (R or H) and 171 (R, Q or H) demonstrated to be of major importance (for recent review, see [16]). Therefore, when investigating the transmissibility to sheep of any non-ovine isolates, it is important to consider a range of host genotypes, to account for potentially variable susceptibility.

This paper presents a study of the transmissibility and characterisation of L-BSE in sheep of various genotypes, and assesses whether the current surveillance requirements would be sufficient to detect and classify such cases if they were to occur in the field.

## Materials and methods

### Animal experimentation

All inoculations were carried out under general anaesthesia and in accordance with the United Kingdom (UK) Animal (Scientific Procedures) Act 1986, under license from the UK Government Home Office (Project licence no: 70/6781). Such license is only granted following approval by the internal Animal and Plant Health Agency (APHA) ethical review process as mandated by the Home Office.

Inoculum (10% w/v brain homogenate in normal saline) was prepared from an Italian L-BSE field case (141387/02), and stored at −80 °C prior to use. TSE negative bovine brain, previously sourced from New Zealand, was used as a negative control inoculum with the kind agreement of the New Zealand authorities.

All recipient sheep were supplied from the Defra New Zealand-derived flock [28] and were 4–6 months old at inoculation. For the primary passage, five sheep of each of five *PRNP* genotypes A_136_F_141_R_154_Q_171_/AFRQ, ARR/ARR, ARQ/ARQ; ARQ/VRQ, VRQ/VRQ [the amino acid at codon 141 (L in wild-type, F when polymorphic) is only indicated when it deviates from the wild type] were inoculated intracerebrally (IC) with 1 mL of inoculum. All sheep were housed for the duration of the study in biosecure accommodation that had never previously housed any TSE affected animals, and was fully cleaned and disinfected (20% Chloros) prior to use. Each pen had separate equipment and personal protective clothing. Animals were handled and observed daily as part of routine husbandry procedures, until clinical disease developed, or until a pre-determined experimental endpoint of approximately 5 years post-inoculation (pi). A further two sheep of each genotype were inoculated with negative control brain material and housed in the same building but separated by a concrete wall so that they only shared the same air space.

Following the identification of the first positive animals in the primary passage study, inoculum for sub-passage was prepared from two positive sheep, one AFRQ/AFRQ and one ARQ/VRQ. These animals were selected to reflect the two slightly different clinical presentations that had been observed in the animals that had succumbed to disease at that time. Each ovine source was used to inoculate five AFRQ/AFRQ and five ARQ/VRQ recipients. Both inoculation groups were housed in separate pens, similar to the primary passage study.

### Clinical monitoring

All sheep were examined neurologically prior to inoculation, then monitored daily during routine husbandry procedures (feeding, bedding changes) and weighed monthly. Weight loss prior to cull was expressed in percent of the previous body weight determined prior to weight loss. Routine clinical examinations [29] were conducted quarterly from 8 months pi (primary passage) and 6 months pi (sub-passage), increasing to weekly for any formal suspect animal. Additional neurological examinations [30] were conducted prior to cull (with the exception of the first culled sheep, which was last examined 2 months prior to death), or if animal husbandry staff noted any signs suggestive of a neurological disease. Inoculated animals were also monitored during the daytime by CCTV.

The clinician was unaware of the genotype of specific challenged animals but the identity of the control group in the primary passage was known because these animals were always examined first.

Clinical end-point was considered to have been reached when sheep displayed progressive abnormalities in sensation (positive scratch test with or without alopecia, absent menace response) and movement (ataxia, limb weakness, tremor). In addition, the display of signs likely to result in permanent recumbency or death, such as seizures or collapse not triggered by human intervention, was considered to be a clinical end-point, which resulted in cull using quinalbarbitone sodium (Somulose, Arnolds) intravenously. Animals, including the controls, that remained healthy throughout the study, were killed at the end of the study. One control animal from each genotype pair was killed after the last animal in the matched genotype challenged group succumbed, and the others were kept until the endpoint of the study, approximately 5 years post challenge.

### Pathology and immunohistochemistry

Postmortem, the whole brain was removed from each sheep and hemisected longitudinally. One half of the brain was placed into 10% formal saline for histology, and the other half stored at −80 °C. Samples representative of the trigeminal, nodose, cranial cervical, stellate and coeliaco-mesenteric ganglia, the lateral retropharyngeal and mesenteric lymph nodes, the recto-anal mucosa-associated lymphoid tissue (RAMALT), the spleen, the distal ileum (with Peyer’s patches) and the extraocular muscles were also collected into 10% buffered formalin.

All brain tissue was routinely fixed, blocked to represent the major levels of the neuraxis and processed, sectioned and stained with haematoxylin and eosin as described in detail elsewhere [31]. Immunohistochemical detection of PrP^Sc^ was performed on adjacent sections from the same brain blocks, and on the other tissues, using mouse monoclonal antibody (mAb) 2G11 (ABD Serotec), raised against the ovine PrP peptide sequence 146-R_154_ R_171_-182, as described in detail elsewhere [32]. Sections from the obex were also immunolabelled with the mAb P4, which can be used to discriminate ovine BSE from scrapie [33]. Vacuolation and immunohistochemistry profiles were created using standard subjective methods as previously described [31, 34] in which the severity of vacuolar lesions, or the type of PrP immunolabelling, is assessed in a standard range of precise neuroanatomical areas. Some modifications were made to the original method [34] to accommodate the range of morphological PrP^Sc^ immunolabelling types first identified in atypical scrapie.

### Western immunoblot

All samples were subjected to the BioRad TeSeE™ Western immunoblot (according to the manufacturer’s instructions) and, run on two replicate gels (18.75 mg tissue equivalent per well). The primary antibodies were Sha31 (prepared according to the kit protocol) and P4 (0.2 µg/mL, RIDA^®^, r-Biopharm). The signal was developed using ECL detection blotting reagents, detected and analysed with exposure times of 1 and 10 min using Biorad Quantity One vers. 4.6.9 software on Fluor-S Max imager. Where Sha31 and P4 antibody ratio calculations were performed the images for both antibodies were obtained at the same time and 1 min exposure times were used.

The original bovine donor animal, a classical bovine BSE field case, an ovine classical scrapie field case, an experimentally transmitted ovine BSE [35] and a negative ovine sample were used as controls alongside the primary passaged samples. The same field case bovine BSE and ovine scrapie controls were used as controls alongside the sub-passaged samples.

Western immunoblots were also performed on retropharyngeal lymph node from the single primary passage pre-clinical VRQ/VRQ animal which displayed peripheral tissue involvement. Brain tissue from the same animal, lymphoid and brain tissue from experimentally transmitted ovine classical BSE and brain tissue from the field case bovine BSE and field case ovine BSE were used as comparative controls.

### Elisa

Samples from all positive animals from the primary passage were subject to four commercially available ELISA tests—IDEXX Herdchek™ with bovine conjugate, IDEXX Herdchek™ with ovine conjugate, BioRad TeSeE™ and BioRad S&G™. For each test, samples were extracted and the tests carried out in accordance with the manufacturers’ instructions.

## Results

Details of all inoculated animals can be found in Table 1. There was a wide range of overlapping incubation periods for each affected genotype in the primary passage.

**Table 1:** **Summary of inoculation outcomes with survival times and rates**

Passage	Recipient genotype	No positive/challenged	Individual survival times (dpi) for positive sheep	Mean ip (±SD) of clinically positive sheep	Mean survival times (+SD) of negative sheep
Primary	VRQ/VRQ	4/5	1183, 1337, 1439,1963^a^,	1320 ± 129	1964
	VRQ/ARQ	5/5	803^b^, 864, 957, 1056, 1389	1014 ± 231	
	ARQ/ARQ	5/5	956, 959, 1186, 1531, 1649	1256 ± 321	
	AFRQ/AFRQ	4/5	1186^b^, 1236, 1267, 1334	1256 ± 62	867^c^
	ARR/ARR	0/5			2001 ± 3
Subpassage: Donor ARQ/VRQ	ARQ/VRQ	5/5	433, 454, 462, 478, 478	461 ± 19	
	AFRQ/AFRQ	5/5	503, 505, 527, 552, 552	528 ± 24	
Subpassage:Donor AFRQ/AFRQ	ARQ/VRQ	5/5	461, 488, 496, 501, 531	495 ± 25	
	AFRQ/AFRQ	5/5	473, 519, 520, 520, 539	514 ± 25	

^a^Preclinical animal, detected at end of study cull (not included in mean survival time calculation).

^b^Animals used as donors for the sub-passage study.

^c^Intercurrent disease loss.

The survival periods for the sheep in the sub-passage study were substantially shorter than those of the donor animals.

Using the two-sample Kolmogorov–Smirnov tests of the equality of distributions, pairwise comparison of the sub-passage groups revealed that the survival period in the ARQ/VRQ homologous transmission group was significantly different to those of the other three groups (ARQ/VRQ donor to AFRQ/AFRQ recipient: *p* = 0.007; AFRQ/AFRQ donor to AFRQ/AFRQ recipient: *p* = 0.05; AFRQ/AFRQ donor to ARQ/VRQ recipient: *p* = 0.05.) The ARQ/VRQ donor into AFRQ/AFRQ recipient group, and the AFRQ/AFRQ donor into the ARQ/VRQ recipient group were also significantly different from each other (*p* = 0.05).

### Clinical observations

The 10 control animals inoculated with normal brain survived to the relevant endpoint of the study; three (one AFRQ/AFRQ, one ARQ/ARQ and one ARQ/VRQ) were killed after the last challenged animal in the relevant challenge group succumbed to clinical disease, at 1678 ± 20 days post challenge (dpi). Neurological signs indicative of a vestibular disease were seen in one sheep (39/13, AFRQ/AFRQ) from 1376 dpi. A positive scratch test [29] was displayed irregularly by seven sheep between 915 and 1681 dpi attributable to hay in the fleece and/or lesions suggestive of *Chorioptes* mange. Neither wool loss nor weight loss was recorded in any of the control animals prior to cull. The remaining seven control animals were culled at 1978 ± 1 dpi.

From the primary passages, only two VRQ/VRQ animals and the five ARR/ARR animals survived to the pre-determined cull point at approximately 5 years post challenge. None of the ARR/ARR sheep displayed signs suggestive of a neurological disease, but both VRQ/VRQ sheep inconsistently displayed fore limb hypermetria and circled when blindfolded, from 1225 and 1713 dpi respectively. Both sheep were considered clinically healthy at cull. At post-mortem, one of the VRQ/VRQ animals (48/13) was found to be positive by all diagnostic methods, while the other clinically normal animals were negative by all tests.

One AFRQ/AFRQ sheep developed changes in behavior and mental status (confusion, separation from others, pica) from 860 dpi and subsequent ataxia, hypermetria, circling and loss of balance when blindfolded, but no weight loss. This sheep was culled at 867 dpi as a TSE suspect, but TSE was not confirmed by postmortem testing.

In the primary passage study, two clinical syndromes were described based on the major presenting clinical sign (Table 2). Pruritic behaviour (rubbing, scratching and nibbling itself) resulting in wool loss and later ataxia, which was indistinguishable from the pruritic form of classical scrapie, or experimental ovine classical BSE [35] was displayed by two sheep. These sheep also presented with a positive scratch test that could later be elicited by merely applying firm pressure on the back (see Additional file 1: pruritic form). More frequently, affected animals presented with a cataplectic form of disease, characterized by collapsing episodes with reduced muscle tone, often with low head carriage and initially triggered by lifting of the head or more stressful events (e.g. foot trimming, restraint for clinical examinations), although in the later stages this occurred during feeding time or in the absence of any obvious stimulus. Initially these episodes were usually very brief, with loss of muscle tone in the limbs causing sheep to drop suddenly to the ground but get up again immediately. With disease progression, complete loss of muscle tone led to lateral recumbency and a complete lack of response, suggestive of narcolepsy (see Additional file 2: cataplectic form). Pruritus was not evident in these cases although some displayed a positive scratch test the significance of which was difficult to interpret given the similar observations in the control sheep.

**Table 2:** **Clinical syndromes in sheep challenged with L-type BSE**

Passage	Animal ID	Genotype	Syndrome	Other neurological and behavioural signs
Primary	456/11	VRQ/VRQ	Cataplectic^a^	None
	113/12		Cataplectic	Ataxia
	167/12		Cataplectic	Head tremor, occasionally horizontal nystagmus during collapsing episodes, teeth grinding
	1591/10^b^	VRQ/ARQ	Undefined, (possibly pruritic)	Seizure-like episode, positive scratch test
	58/11		Cataplectic	Dullness, ataxia, loss of balance
	4/12		Cataplectic	Head tremor, ataxia
	140/11		Cataplectic	Positive scratch test, head tremor, ataxia
	267/11		Cataplectic	Positive scratch test, ataxia
	63/11	ARQ/ARQ	Pruritic	Dullness, head tremor, ataxia, loss of balance
	3/11		Cataplectic	Positive scratch test, ataxia, loss of balance
	182/12		Pruritic	Positive scratch test, ataxia, loss of balance
	3/13		Cataplectic	Dullness, teeth grinding, absent menace response, ataxia, loss of balance
	455/11		Cataplectic	Positive scratch test, ataxia
	98/11^b^	AFRQ/AFRQ	Cataplectic	Ataxia
	457/11		Cataplectic	Head tremor, ataxia
	26/12		Cataplectic	Head tremor, ataxia, loss of balance
	112/12		Cataplectic	Positive scratch test, head tremor, ataxia
Subpassage: donor VRQ/VRQ	112/14	ARQ/ARQ	Cataplectic	Dullness, positive scratch test, absent menace response, head tremor, ataxia, loss of balance, circling
	120/14		Cataplectic	Positive scratch test, absent menace response, head tremor, ataxia, circling, teeth grinding
	119/14		Cataplectic	Positive scratch test, head tremor, ataxia, loss of balance, circling
	107/14		Cataplectic	Absent menace response, head tremor, ataxia, teeth grinding
	108/14		Cataplectic	Head tremor, ataxia, circling
	79/14	ARQ/VRQ	Cataplectic	Positive scratch test, head tremor, loss of balance
	78/14		Cataplectic	Positive scratch test
	76/14		Cataplectic	Head tremor
	6/14		Cataplectic	Ataxia
	73/14		Cataplectic	Absent menace response, head tremor, loss of balance
Subpassage: donor AFRQ/AFRQ	109/14	ARQ/ARQ	Cataplectic	Absent menace response, head tremor
	110/14		Cataplectic	Absent menace response, head tremor, ataxia
	111/14		Cataplectic	Absent menace response, head tremor, ataxia
	118/14		Cataplectic	Absent menace response, head tremor, ataxia
	77/14		Cataplectic	Absent menace response, ataxia
	81/14	ARQ/VRQ	Cataplectic	Head tremor, loss of balance
	80/14		Cataplectic	Head tremor, teeth grinding
	117/14		Cataplectic	Head tremor, ataxia, loss of balance
	82/14		Cataplectic	Head tremor, ataxia, loss of balance, circling

^a^Cataplexy is spontaneous collapse due to complete atonia of skeletal muscles, often associated with narcolepsy (disorder of normal sleep mechanism, usually excessive sleep). The latter may also be a feature in affected L-type BSE-inoculated sheep but is difficult to diagnose.

^b^Animals selected for subpassage.

The clinical classification was equivocal in the first sheep culled with confirmed TSE (1591/10) because it displayed a positive scratch test without any other signs of pruritus at a time when none of the controls showed this sign but was culled after being observed dropping to the floor with limb muscle contraction, similar to a seizure and contrary to the atonia seen in the cataplectic form (see Additional file 3: seizure-like disorder).

Weight loss of 2.1–14.6% (mean 6.8%) prior to cull was recorded in 15 primary passage sheep (88%).

In the subpassage study, one ARQ/VRQ sheep inoculated with the AFRQ/AFRQ donor inoculum was found dead at 461 dpi without any premonitory signs being observed. Minor weight loss (1.7%) was present prior to death. This sheep was confirmed positive by postmortem examination.

All the other subpassage animals, regardless of the donor/recipient combination, presented with the cataplectic syndrome. Five sheep of one inoculation group also displayed a positive scratch test, probably attributable to the chorioptic mange that was diagnosed in this group.

Nine of the 19 subpassage recipients (47%) that succumbed to clinical disease lost weight prior to cull (0.8–20.4%, mean 7.0%). All 19 sheep were positive by all diagnostic methods.

### Pathology

**Figure 1:**
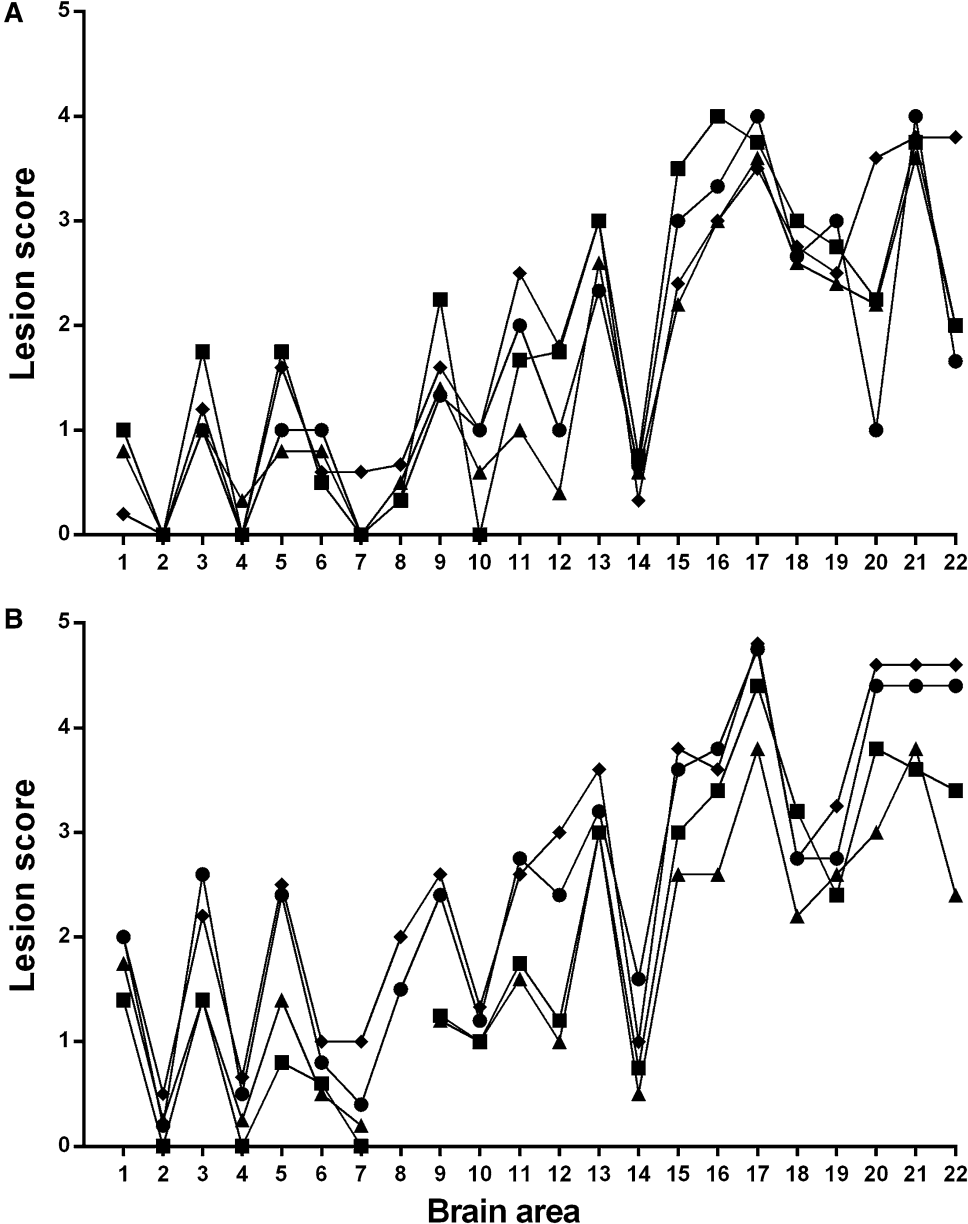
**Vacuolar lesion profiles. A** Primary transmission of bovine L-BSE to sheep. Square = AFRQ/AFRQ recipients (*n* = 4), diamond = ARQ/ARQ recipients (*n* = 5), triangle = ARQ/VRQ recipients (*n* = 5), circle = VRQ/VRQ recipients (*n* = 3). **B** Subpassages of ovine L-BSE to sheep. Square = ARQ/VRQ recipient, ARQ/VRQ donor (n = 5), diamond = AFRQ/AFRQ recipient, AFRQ/AFRQ donor (*n* = 5), triangle = ARQ/VRQ recipient, AFRQ/AFRQ donor (*n* = 5), circle = AFRQ/AFRQ recipient, ARQ/VRQ donor (*n* = 5). X-axis: brain areas from Ligios et al. [31]. 1–11 brainstem areas, 12, 13 the cerebellum, 15–19 midbrain and thalamus, 20–22 the basal ganglia and frontal cortex. Y-axis: mean vacuolation score. Vacuolation is consistently greater in the more rostral brain areas than in the brainstem, regardless of genotype or passage history, with a slight but consistent increase in the intensity of vacuolation throughout the brain following sub-passage.

Vacuolar pathology consistent with TSE was present in all positive cases, with a distribution in the brain that was similar in all L-BSE recipient genotypes (Figure 1) regardless of the clinical presentation or passage history, and distinctly different from animals of the same genotypes infected with classical scrapie or C-BSE [31]. There was limited vacuolation in the brainstem, and the intensity of vacuolation increased markedly in more rostral areas, with high scores for the thalamic areas, the basal ganglia and the cerebral cortex (frontal lobes).

**Figure 2:**
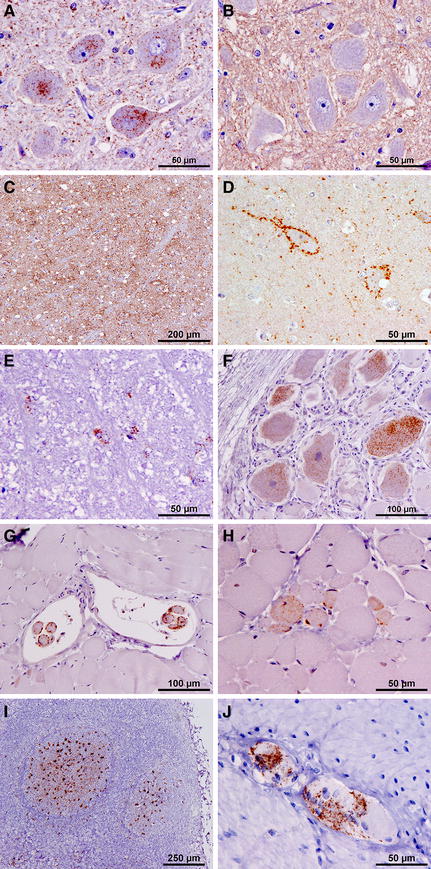
**Immunohistochemistry representative of ovine L-BSE.** All figures illustrate labelling with mAb 2G11, except **B**. Neuronal and particulate labelling is present in the DNV with mAb 2G11 (**A**), but absent with mAb P4 (**B**) (case 455/11). Particulate labelling and small aggregates are abundant in many areas, such as the thalamic nuclei (**C**) (case 1591/10). Perineuronal labelling in the putamen is a consistent and striking feature of ovine L-BSE (**D**) (case 1591/10), as is intracellular labelling of oligodendrocytes, seen here in the spinocerebellar tract (rostral medulla) (**E**) (case 58/11). Intraneuronal labelling is also present in the DRG (**F**) (case 4/12). Heavy labelling in muscle spindles is also visible (**G**) (case 267/11) and also in occasional myocytes (**H**) (case 98/11). Labelling was also present in the LRS (**I**) and ENS (J) of one VRQ/VRQ recipient (case 48/13).

Widespread immunolabelling was present at all levels of the brain and spinal cord with antibody 2G11. In every case, the predominant labelling type was fine particulate in the neuropil (Figure 2), with intraneuronal and glial labelling also present, and some linear forms and small aggregates. Stellate forms were rarely seen. All of the intraneuronal labelling, and most of the neuropil labelling was lost with antibody P4 (Figures 2A and B), which would be diagnostically consistent with a discriminatory interpretation of “BSE-like” [33]. Distinctive perineuronal labelling was seen in the basal ganglia (Figure 2D). Intracellular labelling was also present in the oligodendrocytes.

**Figure 3:**
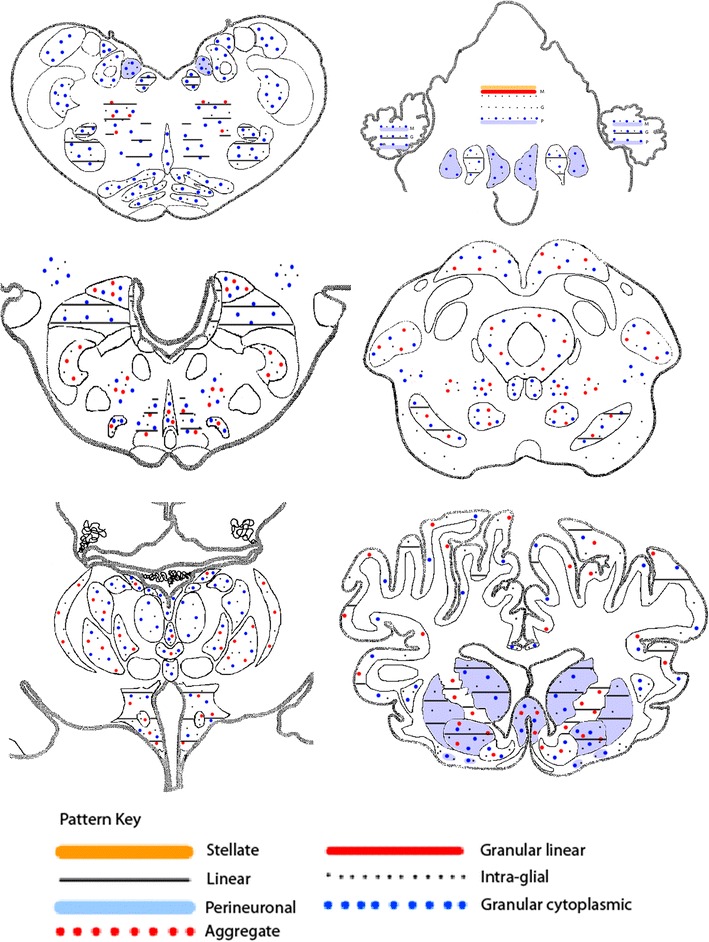
**Schematic non-quantitative representation of the neuroanatomical distribution of PrP immunolabelling.** Every area that was positive contained particulate labelling, so this has been omitted from the figure to make the presence and distribution of the other labelling types easier to see. This map is based on a single case (case 455/11) as representative of all positive animals.

Detailed mapping using 2G11 revealed no obvious difference in immunolabelling patterns between animals of different genotypes or clinical presentation groupings. Figure 3 shows the mapping of labelling type distribution in one case (455/11) as representative of the central nervous system pathology seen in all the positive sheep in this study. The one VRQ/VRQ sheep that was identified as pre-clinically affected at the 5-year cull point (48/13) demonstrated positive immunolabelling throughout the brainstem, but was negative in the cerebellum and cortical regions. The immunolabelling patterns identified in this case were similar to those seen in the other positive animals, but with the addition of stellate forms in several neuroanatomical nuclei.

A range of peripheral tissues from each case was systematically screened by IHC (Table 3). In seventeen of the eighteen positive animals, no immunoreactivity was detected in liver, spleen, lymph nodes, distal ileum or RAMALT. Labelling was consistently seen in the neuronal cell bodies of the trigeminal and nodose ganglia, but not in the cranial cervical or stellate ganglia, or the coeliaco-mesenteric plexi. Labelling of the muscle spindles was seen consistently, and in some cases, occasional labelled myocytes were also observed.

**Table 3:** **Peripheral tissue immunolabelling following primary passage of sheep with L-BSE**

Animal ID	Genotype	Clinical status	Survival time (days)	Ganglia						Lymphoid tissues					Muscle
				Trige-minal	Nodose	Cranial cervical	Stellate	Coeliaco-mesenteric	ENS	LRLN	MLN	RAMALT	Spleen	PP	Extra-ocular
457/11	AFRQ/AFRQ	+	1236	+	+	–	–	–	–	–	–	–	–	–	+^a^
112/12		+	1334	+	+	–	–	–	–	–	–	–	–	–	+
26/12		+	1267	+	+	–	–	–	–	–	–	–	–	–	+
98/11		+	1186	+	+	–	nr	–	–	–	–	–	–	–	+^a^
1606/10		+	867	–	–	–	–	–	–	–	–	–	–	–	–
3/11	ARQ/ARQ	+	959	nr	+	–	–	–	–	–	–	–	–	–	+
182/12		+	1531	+	+	–	–	–	–	–	–	–	–	–	+^a^
63/11		+	956	nr	+	–	nr	–	–	–	–	–	–	–	+
455/11		+	1186	+	+	–	–	–	–	–	–	–	–	–	+^a^
3/13		+	1649	+	+	–	–	–	–	–	–	–	–	–	+^a^
223/13	ARR/ARR	–	1998^b^	–	–	–	–	–	–	–	–	–	–	–	–
224/13		–	1999^b^	nr	nr	–	–	–	–	–	–	–	–	–	–
10/14		–	2005^b^	–	–	–	–	–	–	–	–	–	–	–	–
8/14		–	2000^b^	–	–	–	–	–	–	–	–	–	–	–	–
9/14		–	2005^b^	–	–	–	–	–	–	–	–	–	–	–	–
4/12	ARQ/VRQ	+	1389	+	–	–	–	–	–	–	–	–	–	–	+^a^
140/11		+	957	+	+	+	–	–	–	–	–	–	–	–	+
1591/10		+	803	+	+	–	–	–	–	–	–	nr	–	–	+
267/11		+	1056	nr	+	inc	inc	–	–	–	–	–	–	–	+
58/11		+	864	+	+	–	nr	–	–	–	–	–	–	–	+^a^
225/13	VRQ/VRQ	–	1964^b^	–	nr	–	–	–	–	–	–	–	–	–	–
113/12		+	1337	+	+	–	–	–	–	–	–	–	–	–	+^a^
456/11		+	1183	+	nr	+	–	–	–	–	–	–	–	–	+
48/13		–	1963^b^	nr	nr	–	inc	+	+	+	+	+	+	+	+
167/12		+	1439	+	+	–	–	–	–	–	–	–	–	–	+

nr: not retrieved; inc: inconclusive, ENS: enteric nervous system, distal ileum, LRLN: lateral retropharyngeal lymph node, MLN: mesenteric lymph node, RAMALT: recto-anal mucosa-associated lymphoid tissue, PP: Peyer’s patches of the distal ileum.

^a^Occasional positive myocytes in addition to spindle labelling.

^b^Killed at end of study. No clinical signs.

In the one pre-clinical VRQ/VRQ animal, there was positive labelling present in all the lymphoid tissues examined, together with the enteric nervous system and the coeliaco-mesenteric ganglion in addition to the muscle spindles (Figures 2I and J). Unlike the other cases, labelling with P4 was retained in the brainstem, which would be diagnostically consistent with a discriminatory interpretation of “scrapie-like”. However, P4 labelling was lost in the lymphoid tissue, which would be consistent with a BSE-like interpretation.

The vacuolar profiles (Figure 1B) and immunohistochemical PrP distribution patterns in the sub-passage animals (not illustrated) were the same as those of the donor animals.

No histological lesions or PrP accumulation were detected in any of the control sheep.

### Biochemistry

All the clinical cases from the primary passage tested positive using all four different ELISA formats (Table 4).

**Table 4:** **Comparative ELISA results for primary challenge animals**

	OD (±SD)			
	IDEXX Herdchek BSE/Sc EIA		BioRad	
	SRB-CC^a^	CC^b^	S&G^c^	TeSeE
Ovine L-BSE primary challenge (all genotypes) (*N* = 17^d^)	2.61 (±0.024)	2.62 (±0.031)	2.68 (±0.057)	2.67 (±0.062)
Ovine C-BSE	2.636	2.668	2.631	2.661
Ovine scrapie	2.632	2.672	2.599	2.635
Ovine negative	0.022	0.026	0.029	0.036
Bovine L-BSE	2.630	2.668	0.063	2.617

^a^Small ruminant brain conjugate concentrate.

^b^Bovine brain conjugate concentrate.

^c^Sheep and goat.

^d^Clinical cases only. The pre-clinical VRQ/VRQ is not included.

**Figure 4:**
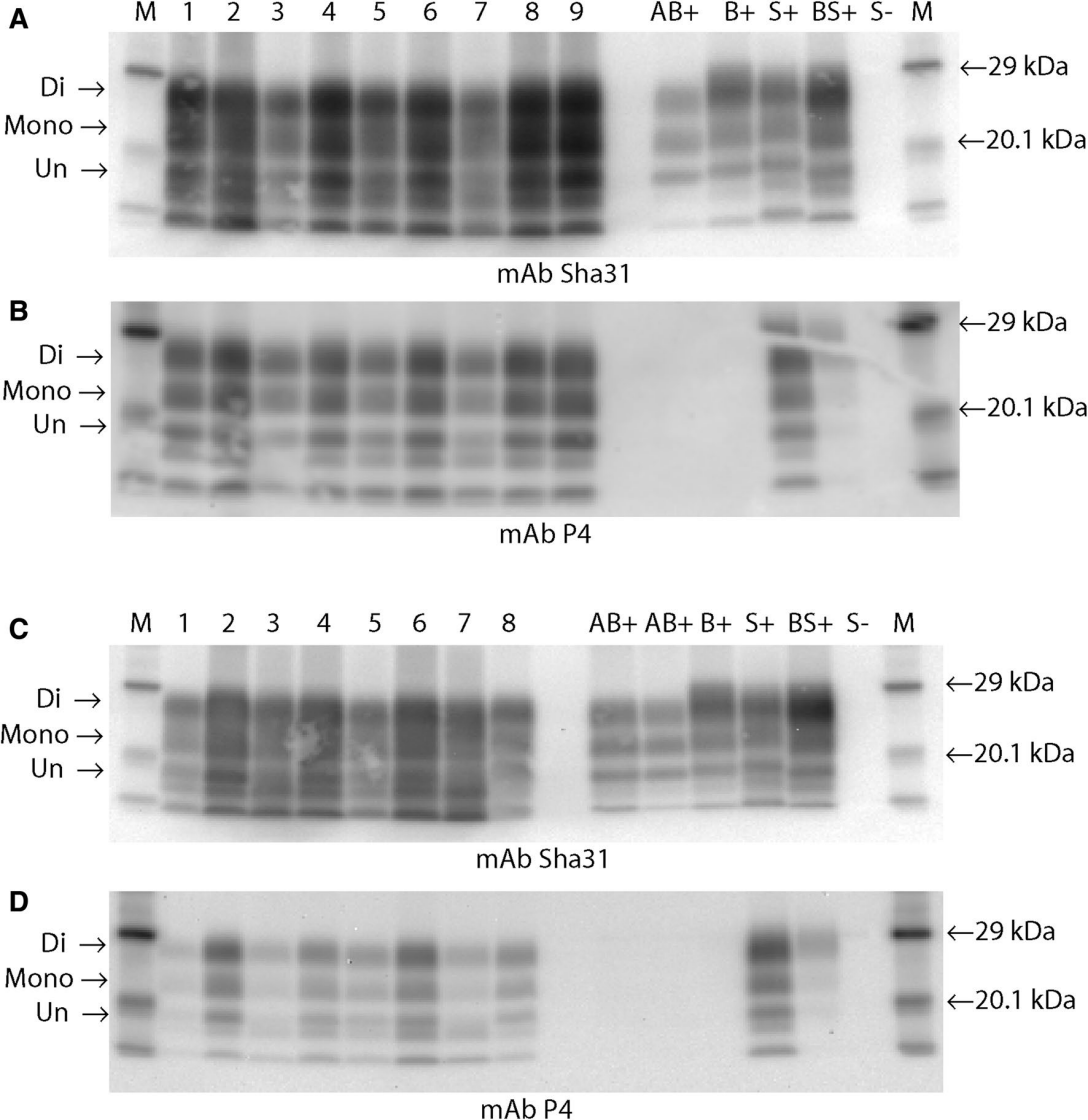
**WB images from primary passage samples. A** and **B** Nine representative recipients, homozygous for alanine (codon 136) detected by Sha31 (**A**), or P4 (**B**). (Lane 1, 63/11; lane 2, 3/11; lane 3, 457/11; lane 4 26/12; lane 5 112/12; lane 6, 182/12; lane 7, 3/13; lane 8, 98/11; lane 9, 455/11; AB + , donor bovine L-BSE; B + , bovine C-BSE; S + , ovine Scrapie; BS + , experimental ovine CBSE; S-, negative sheep; M, molecular mass markers. **A** 1 min exposure, **B** 10 min exposure). **C** and **D** Eight representative recipients homozygous or heterozygous for valine (codon 136), detected by Sha31 (**C**) or P4 (**D**), (Lane 1, case 1591/10; lane 2, case 58/11; lane 3, 140/11; lane 4, 267/11; lane 5, 456/11; lane 6, 113/12; lane 7, 4/12; lane 8, 167/12; markers and controls as for **A** and **B**. **C** 1 min exposure, **D** 10 min exposure). There is low molecular mass migration of the unglycosylated band (arrow), similar to that of the donor bovine L-BSE (AB+), for all but one of the ovine recipients (VRQ/VRQ Lane 8 **C**, **D**) regardless of genotype. Similar di-;mono-glycosylated band ratios are also seen in all cases when detected by mAb Sha31 (see Additional file 4). All recipient samples, irrespective of genotype, are also detected with mAb P4 (**B**, **D**). In contrast the donor L-BSE case (AB+) is not. Following extraction with a Proteinase K digestion step, may contain a mixture of varying molecular mass fragments, partly due to multiple cleavage sites and variability in resistance of PrP^Sc^ to the concentration of the enzyme. The two additional lower bands observed in these profiles are regularly observed in diagnostic samples processed in this way. For diagnostic analysis they are disregarded. Only the standard three bands pertaining to the di, mono and un-glycosylated forms of PrP^Sc^ are considered relevant.

The L-BSE recipients that succumbed to clinical disease were subjected to comparative Western immunoblotting (Figures 4A–D). The samples were grouped according to genotype—those which were ARQ/ARQ, with either F or L at codon 141 (Figures 4A and B) and those which carried one or two VRQ alleles (ARQ/VRQ or VRQ/VRQ) (Figures 4C and D).

The donor profile exhibited the three visual characteristics that are associated with L-BSE: a low molecular mass migration, a glycoform ratio where the di- and mono-glycosylated bands contain a more equal proportion of PrP^Sc^ than classical BSE when detected with the core mAb (Sha31), and a lack of detection with the N-terminal mAb (P4).

**Figure 5:**
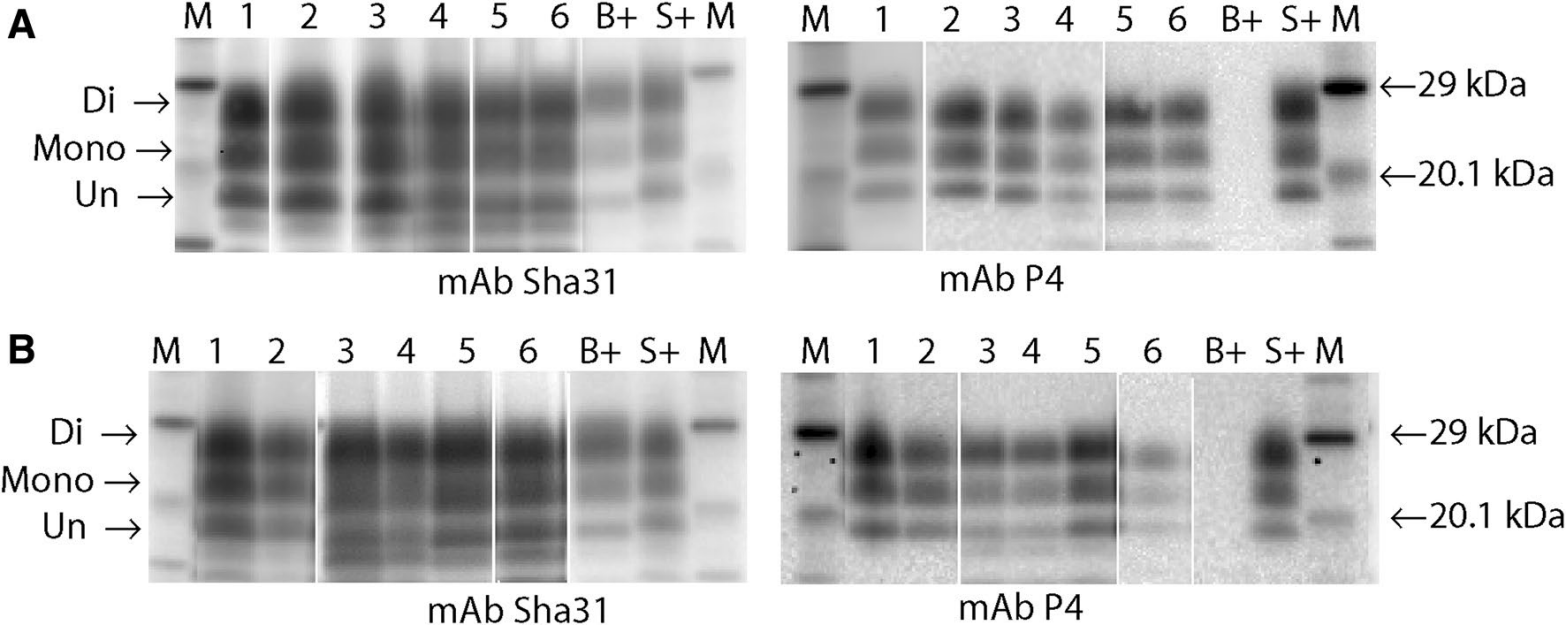
**WB for ARQ/VRQ subpassage recipients. A** Sha31 and P4 blots of the ARQ/VRQ donor and its ARQ/VRQ recipients. (Lane 1, case 1591/10 (ARQ/VRQ Donor), Lane 2, case 6/14; Lane 3, case 73/14; Lane 4, case 76/14 Lane 5, case 78/14; Lane 6, case 79/14 M, Molecular marker; B+, Bovine BSE; S+, Classical Ovine Scrapie. This panel comprises a mixture of 1 and 10 min exposures). **B** Sha31 and P4 blots of the AFRQ/AFRQ donor and its ARQ/VRQ recipients (Lanes 1 and 2, case 98/11 (AFRQ/AFRQ Donor); Lane 3, case 80/14; Lane 4, case 81/14; Lane 5, case 82/14; Lane 6, case 75/14; M, molecular marker; S+, classical ovine scrapie; B+, bovine BSE. This panel comprises a mixture of 1 and 10 min exposures). In contrast the donor L-BSE case (AB+)molecular characteristics described for primary passage (Figure 4; Additional files 4 A–E) are retained on subpassage for all recipient animals regardless of the donor.

In affected animals the overall trend observed was maintenance of the low molecular mass and a glycoform profile similar to L-BSE or classical scrapie, irrespective of genotype (representative cases are illustrated in Figure 4 and Additional file 4), although in one VRQ/VRQ sheep (167/12; Lane 8, Figures 4C and D) the molecular mass migration of the unglycosylated band was slightly higher than for the other cases. There was a substantial increase in the detection by the N-terminal antibody relative to the bovine L-BSE donor, thereby giving an antibody detection ratio for the recipient animals closer to that obtained with classical scrapie. This was more consistent in ARQ/ARQ animals (Figure 4B; Additional file 4) than in those with valine at codon 136. This molecular profile was maintained on sub-passage (Figure 5; Additional file 5).

**Figure 6:**
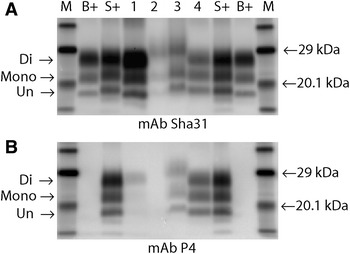
**WB of brain and LRS from 48/13 (the VRQ/VRQ recipient with LRS involvement). A** Detection with mAb Sha31; **B** detection with mAb P4. (M, molecular markers; B+, bovine C-BSE control; S+, ovine classical scrapie control; Lane 1, ovine C-BSE brain; Lane 2, ovine C-BSE lymphoid tissue; Lane 3, ovine L-BSE lymphoid tissue (case 48/13); Lane 4, ovine L-BSE brain (case 48/13). 1 min exposure). The lymphoid tissue from the ovine C-BSE sample (lane 2) and L-BSE recipient (lane 3) show similar but not identical molecular profiles to each other with an overall higher mass migration pattern. However only the L-BSE sample is detected by mAb P4. The ovine C-BSE brain sample exhibits the expected profile characteristics of a low molecular mass migration, predominant diglycosylated band and mimimal detection with mAb P4. The ovine L-BSE brain sample (lane 4) exhibits a molecular mass migration that is higher than expected when compared to the primary and sub passaged results (Figures 4 and 5), appearing similar to the ovine scrapie control. However, the equal intensity of di and monoglycosylated bands can be observed. The sample was also detected by mAb P4.

In two VRQ/VRQ animals the molecular mass of the unglycosylated band was higher than that observed in the other L-BSE samples (Figures 4C (lane 8) and  6). These samples also showed strong affinity with P4. In contrast to all other affected animals, one of these VRQ/VRQ sheep (48/13) had lymphoid tissue involvement showing a molecular profile with overall higher molecular mass for each band compared to the brain samples and was similar, but not identical, to the profile observed in the LRS of sheep affected with classical BSE (Figure 6; Additional file 6). However, strong detection with mAb P4 was still evident in contrast to ovine classical BSE where mAb P4 detection was absent (lymphoid tissue) or markedly reduced (brain tissue). On this blot, the brain tissue from the L-BSE case had a molecular mass migration similar to the classical scrapie control when detected with Sha31.

## Discussion

L-BSE can transmit experimentally, generally by the intracerebral route, to a wide range of hosts. These include cattle [36–39], sheep [40–42] and this report, lemurs [43], macaques [23, 24], hamsters [25, 44, 45] and transgenic mice overexpressing bovine, ovine or human PrP [20, 25–27, 39, 42, 44, 46–48]. In contrast to classical BSE, L-BSE does not transmit to wild type [20] Spiropoulos and Simmons, unpublished data] or transgenic mice that overexpress murine PrP [45, 47], although exceptions have been reported [20].

There is evidence that, unlike classical BSE [49], L-BSE [20, 25, 42, 44, 45, 47], and H-BSE [21, 47, 50] do not always remain stable after intra- or inter-species experimental transmission. In some of these transmissions a phenotype shift from both L-BSE and H-BSE towards C-BSE has been reported [42, 47], on occasions only after sub-passage [25, 44, 45]. Such phenotype shifts have also been observed with other animal TSEs [46, 51].

The present study suggests that L-BSE remains stable on serial passage through sheep with a partial phenotype shift, associated with overt lymphoreticular involvement, observed only in one animal. The observation of a BSE-like labelling pattern in the LRS of this sheep, together with the biosecurity measures that were in place, and the absence of similar observations in any of the other VRQ/VRQ sheep, including the two genotype matched controls, would argue against the otherwise “scrapie-like” phenotype in this sheep being attributable to infection with extraneous classical scrapie.

The disease phenotype observed after intracerebral inoculation of sheep with L-BSE does not resemble any previously known TSE in this species, but is very consistent regardless of ovine host PrP genotype. The two animals selected for sub-passage were chosen because of differences in clinical presentation, most notably that one (1591/10) was positive on the scratch test, while the other one (98/11) was not. This was not in itself sufficient for them to be considered as different phenotypes, and all other phenotypic parameters in these animals were the same, but they represented the only diversity seen at that time in the study. The predominant clinical cataplectic syndrome reported in the primary challenge animals was present in all of the animals challenged in the subpassage study, regardless of which donor was used. All other phenotypic characteristics were also the same, indicating the presence of a single strain, stable on subpassage.

The substantial reduction in survival time between the primary challenges and the subpassages is indicative of an initial transmission barrier between cattle and sheep. In the absence of any other phenotypic differences the differences in survival time between the different recipient genotype groups following subpassage is most likely an effect of host genotype.

There is no data available on whether this species adaptation might alter the host range or virulence of the isolate, as has been reported previously for classical BSE [52–55]. Previous reports on the transmission of L-BSE to sheep have only described the inoculation of animals that are ARQ/ARQ [40, 41] or ARQ/ARR [41]. This present study extends the range of sheep genotypes inoculated experimentally, to include the VRQ, and AFRQ haplotypes. In contrast to the unusual but consistent clinical signs reported here, no specific neurological signs have been described in the other studies.

Certain phenotypic parameters, such as survival times, vacuolation profiles and PrP^Sc^ distribution patterns are not directly comparable between different species. However, Western blots are more specific to each TSE agent and are generally less affected by the host species.

In this study the WB profiles indicate that the molecular mass migration and glycoform ratio of the L-BSE donor isolate remained unchanged after passage in ARQ/ARQ sheep, regardless of the codon 141 polymorphism, in agreement with those reported for other ARQ/ARQ L-BSE ovine challenges. In addition we have observed an increased reactivity with the N-terminal antibody which is not reported in other ovine L-BSE transmissions [40, 41]. This particular characteristic, normally associated with classical scrapie, is also observed on transmission of classical BSE to sheep but seen as a more gradual N-terminal antibody detection that increases after serial subpassage [56]. It is possible that on the cross species transmission the atypical forms of BSE drive conversion of host PrP^c^ to a form more closely resembling classical scrapie faster than classical BSE does.

Other host genotypes result in more variable Western blot characteristics, as demonstrated by the molecular mass migration shift towards a scrapie profile seen in two of the four VRQ/VRQ animals in this study and in one of the ARQ/ARR animals reported elsewhere [41].

Therefore it can be assumed that the species dependent phenotypic parameters, with the possible exception of survival time, which may be prolonged during interspecies transmission as a result of the transmission barrier, are specific for the L-BSE phenotype in sheep. However, concrete evidence that the strain identity has not changed during passage of the agent in sheep can be obtained by comparing bovine and ovine L-BSE sources after bioassay in mice. These bioassays are currently ongoing and will be reported separately.

There is an apparent susceptibility effect of host genotype which mirrors that seen for ovine C-BSE, with ARR/ARR sheep appearing most resistant, and with longer incubation periods associated with VRQ/VRQ animals [57–59]. This is not surprising as ovine PrP polymorphisms play an important role not only in classical scrapie, where their effect on susceptibility was first identified, but also in atypical or Nor98 scrapie. It is reasonable to assume that PrP polymorphisms in sheep would influence susceptibility, and in some cases phenotype, to almost any TSE exposure/challenge in these species.

The absence of detectable PrP in lymphoid tissues in 37/38 positive animals, and the involvement of peripheral ganglia and muscle spindles concurs with the peripheral tissue distribution of L-BSE in cattle. This differs from the widespread lymphoreticular involvement seen in sheep challenged with C-BSE either orally [58] or following intracerebral inoculation ([60], Simmons, unpublished observations) and suggests that L-BSE affected animals are unlikely to readily disseminate the agent in the environment.

The identification of LRS involvement in one inoculated VRQ/VRQ animal, in which there was also stellate PrP deposition in the brain, might suggest the emergence of a new phenotype as a result of this interspecies transmission, or a stochastic event. To resolve this issue, mouse bioassays are ongoing to compare the biological phenotype of this isolate relative to the other inoculated animals. In this study, differences in WB characteristics were also observed between the CNS and LRS from this animal and the ovine C-BSE control. However, this observation is consistent with previous reports for classical scrapie in which it has been speculated that this reflects tissue-specific differences in glycosylation [61, 62].

Pruritus and weight loss are common features in sheep experimentally infected with classical BSE [35]. Although weight loss was also recorded frequently in sheep infected with L-BSE, clear evidence of pruritus was only seen in two ARQ/ARQ sheep whilst other sheep displayed the cataplectic form despite having the same prion protein genotype and being inoculated with the same inoculum. We have previously described similar divergent clinical syndromes in cattle [38, 63]. Testing the response to scratching as an indicator of pruritus was less useful in this study because a response was also seen in control animals and a positive scratch test was only considered to be indicative of pruritus if it was progressive (e.g. mere pressure on the back elicited a response) and accompanied by wool loss.

The display of cataplexy, defined as sudden and transient episodes of loss of motor tone, (often subsequent to intense emotion in humans) in most of the sheep, sometimes accompanied by apparent narcolepsy, was unusual because it preceded other neurological signs suggestive of a TSE, e.g. ataxia or tremor. Although collapsing episodes have been reported in classical scrapie, these are usually seen infrequently and often at late-stage disease, when other signs associated with scrapie (e.g. pruritus, abnormal behaviour) are already present. There are only a few reports of collapsing episodes in scrapie-affected sheep, triggered by stress [64–66] but they are usually accompanied by pruritus, and not described as cataplexy-narcolepsy-like. There is a single report of cataplexy-narcolepsy in a lamb, which was associated with possibly impaired hypocretin function although the cause was not determined [67]. When initially presented with this sign in the first sheep, alternative diagnoses such as cardiac or neuromuscular disorders were considered but excluded based on the absence of abnormalities in heart rate, rhythm and electrocardiogram and the abrupt onset of and recovery from the collapse.

Given the unusual clinical presentation of these animals, any such cases occurring naturally would be unlikely to present as TSE suspects. From a surveillance perspective, we would therefore be reliant on the rapid screening tests applied to fallen stock and/or healthy slaughter populations to detect such cases should they arise. It is reassuring that ovine L-BSE presents with positive test results using all currently approved screening and confirmatory tests, indicating that such cases would be detected as positive within routine active surveillance activities. It is also reassuring that the existing confirmatory and discriminatory tests should identify these cases as unusual, and in some regards BSE-like in presentation, and they should result in referral for further investigation. However, caution should be exercised if Western immunoblot is the only confirmatory test applied since it has been observed here that a low molecular mass suggestive of L-BSE may not always be observed. As these cases are also strongly detected by the N-terminal antibody they could present as very similar to scrapie in a field situation. For diagnostic purposes, when TSEs in small ruminants present a Western immunoblot profile that contains mixed characteristics of BSE and scrapie they are more readily visually identified as unusual when detection with an N-terminal antibody is reduced or absent. Otherwise it can be more difficult to determine subtle differences in glycoform ratios and molecular mass migration, particularly when the overall signal strength is high.

No cases presenting with such phenotypic characteristics have been identified to date through the EU surveillance systems. However, given the growing trend to view and treat atypical BSE differently from classical BSE, it is important to know that L-BSE in sheep could be detected, should relaxation of the current preventative measures applied to the ruminant feed chain occur in the future.

## Additional files

**Additional file 1.** **Pruritic syndrome.** Case 63/11 (ARQ/ARQ). This clip demonstrates progression in pruritic activity. Initially at 690 dpi there are some head movements in response to scratching (inconclusive scratch test response). The scratch test is clearly positive at 865 dpi when the sheep responded to scratching with lip licking and nibbling. There are no obvious gait abnormalities at this stage. Pruritic activity, such as rubbing and nibbling self, can be observed subsequently on CCTV from 840 dpi, which culminates in wool loss next to the tail head, indicated in the inset picture. Hind limb ataxia is now present at 949 dpi, which is most obvious when the sheep walks towards the other sheep, the wool loss subsequently remained but no skin lesions developed. Prior to cull at 956 dpi, a positive scratch test can merely be elicited by applying pressure on the back.

**Additional file 2.** **Cataplectic syndrome.** Case 3/11 (ARQ/ARQ). When all sheep are penned up for spray marking, this sheep is seen collapsing briefly when restrained at 924 dpi. A similar response is elicited when the handler attempts to catch the sheep and although the animal is getting up immediately, general ataxia and low head carriage with apparent loss of muscle strength in the neck (“floppy neck”) is evident. CCTV images show lack of muscle tone when nudged by other sheep at the hay rack at 954 dpi. Scratching of the back result in lip and head movements (positive scratch test) although there is no evidence of wool loss. Following a collapse after the examination, the sheep is seen lying in the corner as if asleep and rises shortly after stimulated by hand clapping.

**Additional file 3.** **Seizure-like syndrome.** Case 1591/10 (VRQ/ARQ). The sheep displays a positive scratch test at 746 dpi, the last examination prior to cull. At 803 dpi it is found dropping to the floor, and after walking slowly around the pen division it suddenly runs forward and drops to the floor, briefly vocalising, to end up in lateral position with all four limbs extended and trembling whilst the head is held between the front limbs. This episode only lasts a few seconds and the sheep gets up and runs away but drops down again at the edge of the pen division. Although the animal recovered from this episode it was culled on the same day.

**Additional file 4.** **Antibody and glycoform ratios and molecular masses based on WB in Figure** **4.** A) Antibody detection ratios [Sha31 (Figure 4A): P4 (Figure 4B)] for L-BSE in animals that are homozygous for alanine at codon 136 (samples 1-9). It can be seen that the ratios in all of the challenged animals are very similar to that obtained from the classical scrapie control. Ratios calculated using the 1 min exposure for both antibodies. B) Antibody detection ratios [Sha31 (Figure 4C): P4 (Figure 4D)] for L-BSE in animals that are homozygous or heterozygous for valine at codon 136 (samples 1-8). The ratios in all of the challenged animals are generally higher than those in animals homozygous for alanine at codon 136 (A) but lower than that obtained from any of the bovine samples, with variable values sitting between the ovine classical scrapie and ovine BSE controls. This variability was not attributable to the codon 136 polymorphism, with samples 5, 6 and 8 being the homozygous animals. C) The glycoform profiles (relative quantity of the mono- versus di-glycosylated bands as observed in Figures 4A and 4C, using mAb Sha31). It can be seen that all of the sheep challenged with L-BSE have profiles which cluster with that of the bovine L-BSE donor, while bovine and ovine classical BSE are separate. D) Molecular masses for Sha31 based on Figure 4A. E) Molecular masses for Sha31 based on Figure 4C.

**Additional file 5.** **Antibody and glycoform ratios and molecular masses based on WB in Figure** **5.** A) Antibody detection ratios (Sha31 and P4 (Figure 5A)). All ratios are calculated using the 1 min exposure for both antibodies. The codon 136 alanine/valine heterozygous ovine L-BSE donor (sample 1) and similarly heterozygous recipients (samples 2-6) are variable, but consistently lower than that for ovine classical BSE, and higher than the classical scrapie control. B) Antibody detection ratios [Sha31 and P4 (Figure 5B)]. All ratios are calculated using the 1 min exposure for both antibodies. The codon 136 alanine homozygous ovine L-BSE donor (sample 1) and alanine/valine heterozygous recipients (samples 2-6) are variable, but consistently lower than that for ovine classical BSE, and higher than the classical scrapie control. C) The glycoform profiles (relative quantity of the mono- versus di-glycosylated bands as observed in Figure 5A, using mAb Sha31). All the donor and recipient animals cluster with the classical scrapie control, and away from the ovine classical BSE. D) The glycoform profiles (relative quantity of the mono- versus di-glycosylated bands as observed in Figure 5B, using mAb Sha31). All the donor and recipient animals cluster with the classical scrapie control, and away from the ovine classical BSE. E) Molecular masses for Sha31 based on Figure 5A. F) Molecular masses for Sha31 based on Figure 5B.

**Additional file 6.** **Antibody and glycoform ratios and molecular masses based on WB in Figure** **6.** A) Antibody detection ratios (Sha31 (Figure 6A): P4 (Figure 6B)) show that the ovine BSE control samples (samples 1 (brain) and 2 (lymphoid tissue)) match the classical BSE control, whereas the lymphoid tissue (sample 3) and brain (sample 4) from the L-BSE challenged animal show ratios closer to that of the classical scrapie control. All ratios are calculated using the 1 min exposure for both antibodies. B) The glycoform profiles (relative quantity of the mono- versus di-glycosylated bands as observed in Figure 6A, using mAb Sha31) demonstrate that the ovine L-BSE samples (3 and 4) are quite distinct from both the ovine classical BSE samples (1 and 2) and the classical scrapie controls. C) Molecular masses for Sha31 based on Figure 6A.
