# Supplementary figures and images for: L-BSE experimentally transmitted to sheep presents as a unique disease phenotype
Source: Vet Res. 2016 Nov 8;47:112. doi: 10.1186/s13567-016-0394-1 (PMC5101820; doi:10.1186/s13567-016-0394-1)

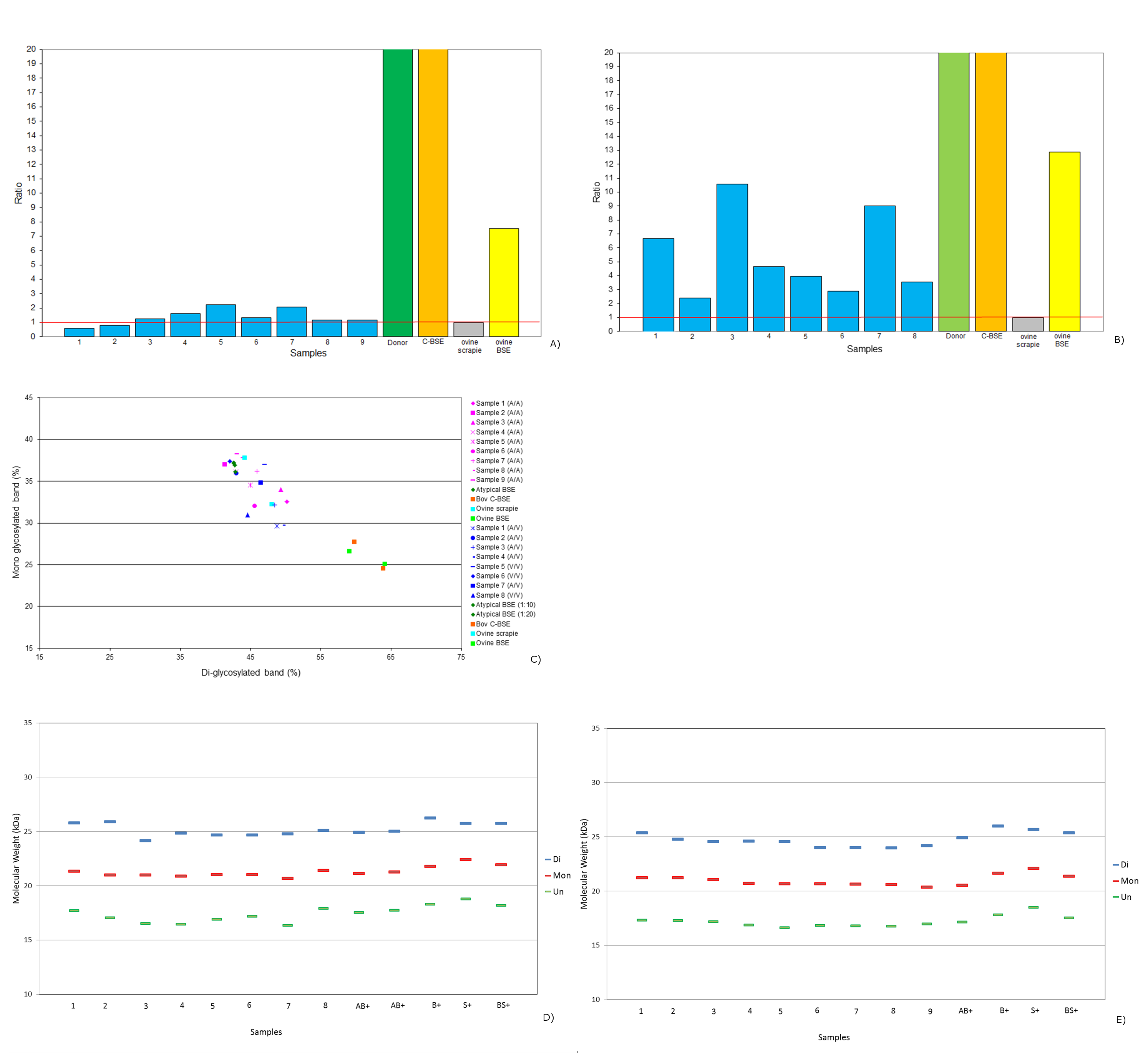

Supplement: Supplementary file 4 — Additional file 4. Antibody and glycoform ratios and molecular masses based on WB in Figure 4. A) Antibody detection ratios [Sha31 (Figure 4A): P4 (Figure 4B)] for L-BSE in animals that are homozygous for alanine at codon 136 (samples 1-9). It can be seen that the ratios in all of the challenged animals are very similar to that obtained from the classical scrapie control. Ratios calculated using the 1 min exposure for both antibodies. B) Antibody detection ratios [Sha31 (Figure 4C): P4 (Figure 4D)] for L-BSE in animals that are homozygous or heterozygous for valine at codon 136 (samples 1-8). The ratios in all of the challenged animals are generally higher than those in animals homozygous for alanine at codon 136 (A) but lower than that obtained from any of the bovine samples, with variable values sitting between the ovine classical scrapie and ovine BSE controls. This variability was not attributable to the codon 136 polymorphism, with samples 5, 6 and 8 being the homozygous animals. C) The glycoform profiles (relative quantity of the mono- versus di-glycosylated bands as observed in Figures 4A and 4C, using mAb Sha31). It can be seen that all of the sheep challenged with L-BSE have profiles which cluster with that of the bovine L-BSE donor, while bovine and ovine classical BSE are separate. D) Molecular masses for Sha31 based on Figure 4A. E) Molecular masses for Sha31 based on Figure 4C. [file 13567_2016_394_MOESM4_ESM.tiff]

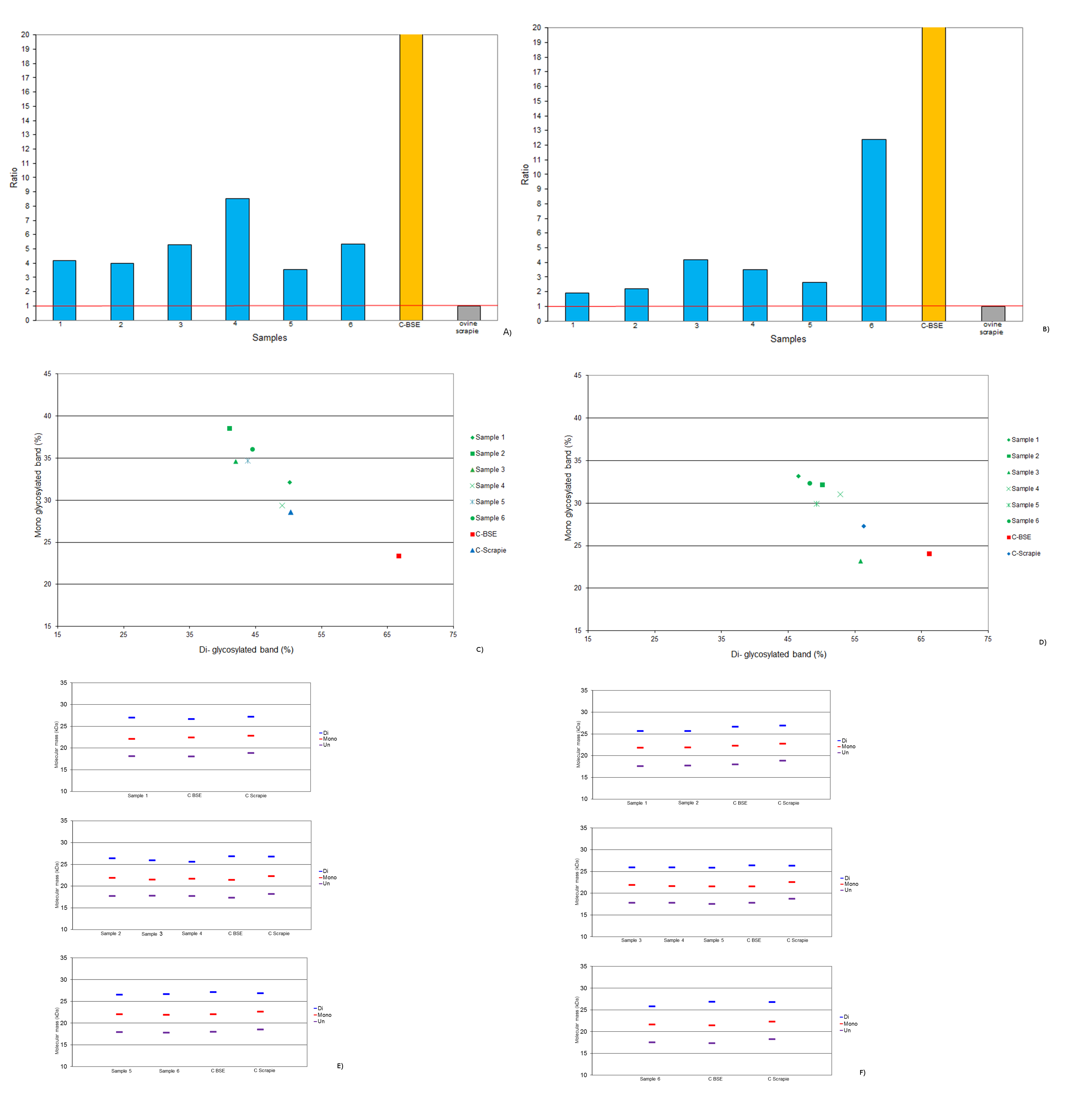

Supplement: Supplementary file 5 — Additional file 5. Antibody and glycoform ratios and molecular masses based on WB in Figure 5. A) Antibody detection ratios (Sha31 and P4 (Figure 5A)). All ratios are calculated using the 1 min exposure for both antibodies. The codon 136 alanine/valine heterozygous ovine L-BSE donor (sample 1) and similarly heterozygous recipients (samples 2-6) are variable, but consistently lower than that for ovine classical BSE, and higher than the classical scrapie control. B) Antibody detection ratios [Sha31 and P4 (Figure 5B)]. All ratios are calculated using the 1 min exposure for both antibodies. The codon 136 alanine homozygous ovine L-BSE donor (sample 1) and alanine/valine heterozygous recipients (samples 2-6) are variable, but consistently lower than that for ovine classical BSE, and higher than the classical scrapie control. C) The glycoform profiles (relative quantity of the mono- versus di-glycosylated bands as observed in Figure 5A, using mAb Sha31). All the donor and recipient animals cluster with the classical scrapie control, and away from the ovine classical BSE. D) The glycoform profiles (relative quantity of the mono- versus di-glycosylated bands as observed in Figure 5B, using mAb Sha31). All the donor and recipient animals cluster with the classical scrapie control, and away from the ovine classical BSE. E) Molecular masses for Sha31 based on Figure 5A. F) Molecular masses for Sha31 based on Figure 5B. [file 13567_2016_394_MOESM5_ESM.tiff]

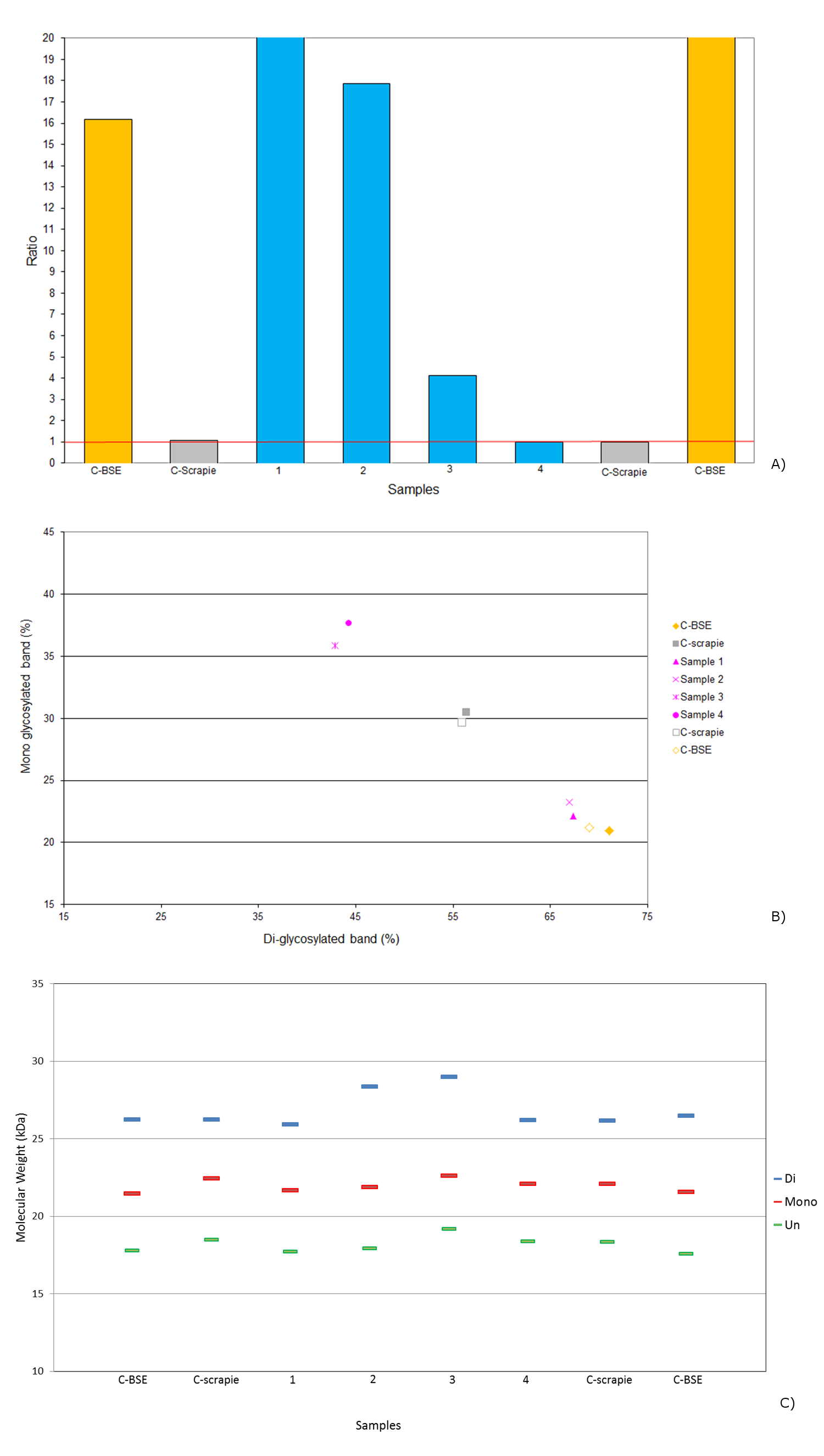

Supplement: Supplementary file 6 — Additional file 6. Antibody and glycoform ratios and molecular masses based on WB in Figure 6. A) Antibody detection ratios (Sha31 (Figure 6A): P4 (Figure 6B)) show that the ovine BSE control samples (samples 1 (brain) and 2 (lymphoid tissue)) match the classical BSE control, whereas the lymphoid tissue (sample 3) and brain (sample 4) from the L-BSE challenged animal show ratios closer to that of the classical scrapie control. All ratios are calculated using the 1 min exposure for both antibodies. B) The glycoform profiles (relative quantity of the mono- versus di-glycosylated bands as observed in Figure 6A, using mAb Sha31) demonstrate that the ovine L-BSE samples (3 and 4) are quite distinct from both the ovine classical BSE samples (1 and 2) and the classical scrapie controls. C) Molecular masses for Sha31 based on Figure 6A. [file 13567_2016_394_MOESM6_ESM.tiff]
